# Successful sequential application of CAR T-cell-therapies in relapsed refractory multiple myeloma

**DOI:** 10.1007/s00277-025-06340-y

**Published:** 2025-04-03

**Authors:** Paul Mobascher, Monika Engelhardt, Ralph Wäsch

**Affiliations:** Department of Hematology, Oncology and Stem Cell Transplantation, Faculty of Medicine, Medical Center - University of Freiburg, University of Freiburg, Hugstetterstrasse 55, 79106 Freiburg, Germany

**Keywords:** CAR T-cells, Cilta-cel, Ide-cel, Sequential use, Extramedullary disease, Multiple myeloma

## Abstract

We here report on a patient with relapsed-refractory multiple myeloma (RRMM) who received chimeric antigen receptor (CAR) T-cells (Ciltacabtagene Autoleucel, cilta-cel, Carvykti^®^) after achieving a partial but not durable remission with Idecabtagene Vicleucel (ide-cel, Abecma^®^). Both CAR T-cells target the B-cell maturation antigen (BCMA) and their sequential use is as yet rare, thus data on safety and efficacy of their sequential employment are precious and relevant for the myeloma commmunity.

Our now 59-year-old patient was diagnosed with lambda light chain (LC) MM in 10/2015, suffering from hypercalcemia and diffuse osteolyses of the left acetabulum, ilium, sternum, thoracic spine, and scapulae (2/4 CRAB criteria). Bone marrow (BM) revealed 30% plasma cells and FISH-cytogenetics were unfavorable (+ 1q, del(13q14)), with ISS 1 and R-ISS 2. He had no comorbidities. He was enrolled in the DSMM XIV trial and received VRd-induction (bortezomib, lenalidomide, dexamethasone). Due to a severe lenalidomide induced skin reaction, lenalidomide was replaced by cylophosphamide. Within this trial, he received an autologous stem cell transplantation and pelvic surgery with radiotherapy consolidation of the left acetabulum and thoracic vertebrae leading to complete remission (CR) by 12/2016. Because of high-risk cytogenetics and according to DSMM/GMMG trials [1, 2], he received bortezomib-maintenance (Vd), until immunofixation and whole-body computer tomography (CT) revealed disease progression (PD) in January 2019. He underwent second-line therapy (2nd LT) with daratumumab, carfilzomib, dexamethasone (Fig. 1A) according to Candor [3] and further radiation therapy. Therewith, he remained stable (SD) until June 2021, when a follow-up whole-body CT again revealed progressive osteolyses. As 3rd LT, the patient received daratumumab combined with the bispecific T-cell engager talquetamab targeting GPRC5D (G-protein-coupled receptor family C group 5 member D) [4]. Unfortunately, treatment had to be discontinued after two cycles due to non-responsiveness. During the short course of talquetamab the patient developed skin rash, skin exfoliation at palms and soles, mucositis, dysgeusia, all CTCAE grade 2.

**Fig. 1:**
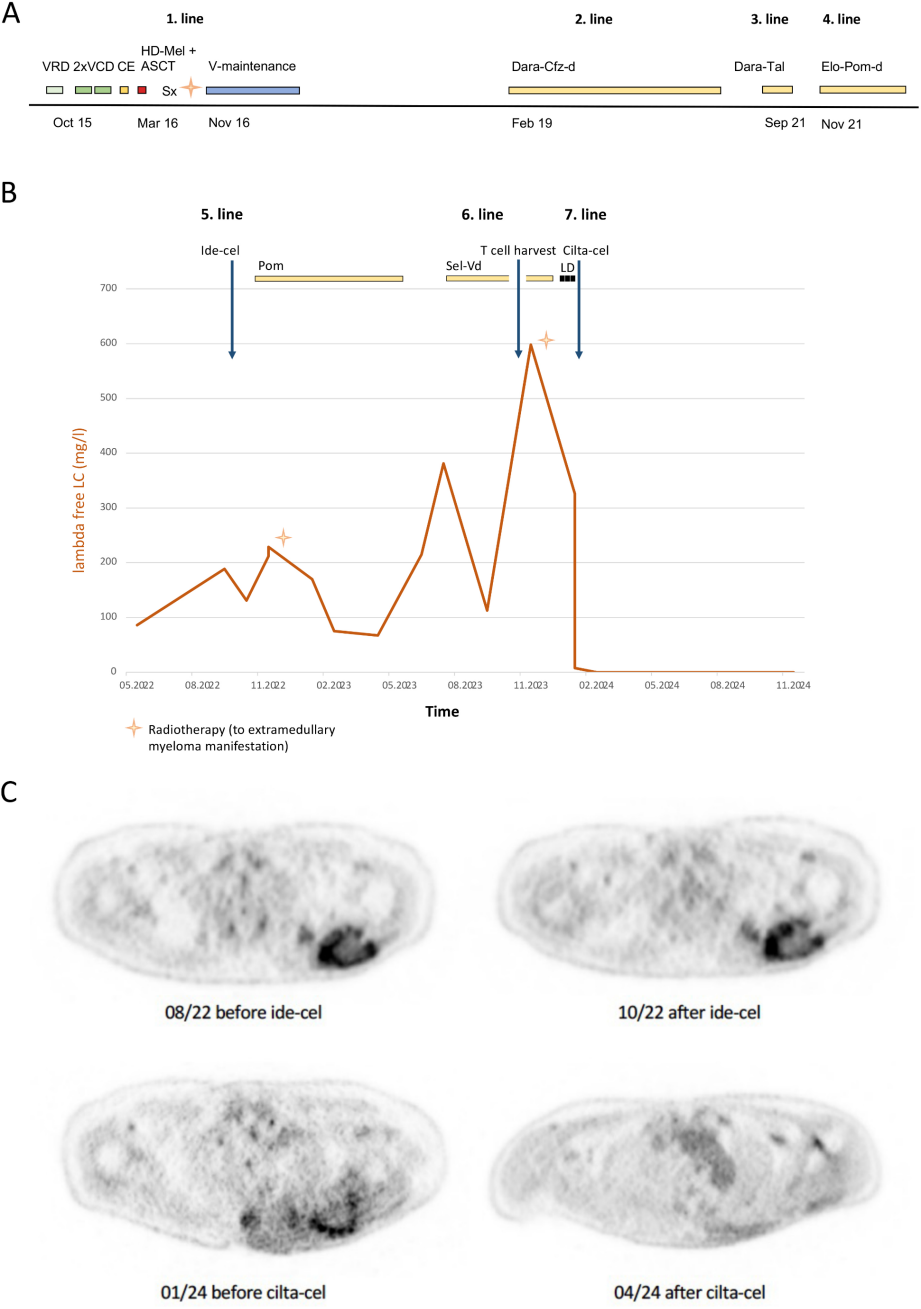
(**A**) Therapeutic timeline prior to first CAR T-cell application. (**B**) Lambda SFLCs showing favorable outcome of cilta-cel after insufficient response to ide-cel and SVd. (**C**) [^18^F]FDG PET/CT before and after ide-cel and cilta-cel to monitor regression of the large lesion of the left scapula. The color scheme for 1st line treatment is green for induction cycles, yellow for mobilization chemotherapy, red for high dose chemotherapy with Mel 200 mg/m^3^ and autologous stem cell transplantation and blue for bortezomib maintenance, for 2nd to 6th line treatment yellow, and for lymphodepletion yellow. Abbreviations: V, bortezomib; C, cyclophosphamide; D, d, dexamethasone; E, etoposide; HD-Mel, high dose chemotherapy with Mel 200 mg/m^3^; ASCT, autologous stem cell transplantation; Sx, surgery; Dara, daratumumab; Cfz, carfilzomib; Tal, talquetamab; Elo, elotuzumab; Pom, pomalidomide; Sel, Selinexor; LD; lymphodepletion, LC, light chains

He transitioned to 4th LT with elotuzumab, pomalidomide, and dexamethasone [5]. Due to triple-class-exposed (TCE) and -refractory (TCR) disease in 06/2022, our multidisciplinary MM-tumor board (MM-TB) [6] discussed CAR T-cell-therapy with ide-cel being the first available in Germany as 5th LT. Upon PD in 09/2022, lymphodepletion with fludarabine and cyclophosphamide (Flu/Cy) was performed and ide-cel given (Fig. 1B) which was well tolerated with neither cytokine release syndrome (CRS) nor immune effector cell-associated neurotoxicity syndrome (ICANS). Unfortunately, 3 months after ide-cel, serological PD was already apparent (lambda serum free LC (SFLC) increase from 131 to 212 mg/l). Pomalidomide (4 mg/day) was added and combined with radiotherapy to the paraskeletal soft-tissue plasmacytoma lesion of the left scapula. In July 2023, 10 months after ide-cel, lambda SFLCs further increased to 381 mg/l (Fig. 1B). The patient received 6th LT with selinexor-Vd (Sel-Vd) as treatment. The lambda light chains initialy decreased to 113 mg/l (PR). Despite this, his paraskeletal MM manifestation worsened in the left scapula with an additional painful lesion at the right tibia, with light chains increasing again to 598 mg/l, and re-irradiation was performed in combination with Sel-Vd as bridging therapy to regain disease control. The MM-TB recommended to proceed with cilta-cel CAR T-cells, considering its superior efficacy compared to ide-cel, and despite the prior weak response to the latter. T-cell harvest was performed, collecting 8.9 × 10⁶ CD3 + cells/kg body weight. Before cilta-cel infusion laboratory results showed lambda SFLCs of 326 mg/L and PET-CT confirmed disease progression (Fig. 1B, C).

After lymphodepletion with Flu/Cy, our patient received his 2. CAR T-cell infusion with cilta-cel as 7th LT. He developed CRS grade 2, which quickly improved under tocilizumab. No ICANS occurred. Following treatment with cilta-cel, CAR-T-cells persisted for 10 months with peak expansion on day 14 with 2704/µl BCMA-CAR-T-cells. B cells are completely depleted until now and there is also ongoing cytopenia without need for growth factor support (grade 2–3 thrombocytopenia, grade 1 neutropenia and anemia). As of February 2025, 13 months after Cilta-cel application, the patient remains in sCR with unmeasurable light chains, negative immunofixation in serum and urine, no malignant plasma cells in his BM and regression of metabolic activity of the large scapula lesion monitored by [^18^F]FDG PET/CT (Fig. 1B, C).

Our case demonstrates that a second BCMA-targeted CAR T-cell therapy can achieve favorable outcomes in RRMM, even after prior immunotherapeutic treatment with bispecific antibodies (here GPRC5D). Unfortunately, we could not reliably assess BCMA-expression prior cilta-cel - in the BM aspirate before cilta-cel there were only 0.1% CD138 + plasma cells with lambda light chain restriction detected by flow potentially due to sampling error. These cells partially coexpressed BCMA - however the striking response ensured sustained expression. Notably, the quality of the patient’s T-cells is ought to influence the response to both talquetamab and cilta-cel. Nonetheless, only the latter proved to be highly effective in contrast to talquetamab, indicating that in this patient, BCMA-CAR Ts via cilta-cel provided a better target than GPRC5D. Sequencing of immunotherapies should consider antigen switching as it has been done here with using ide-cel after talquetamab. Sequential therapy against the same target seems to be most efficient when using different CAR T cell products or CAR T cells before bispecific antibodies, although this has been investigated only in smaller patient cohorts [7–9]. Antigen loss can hamper efficacy, but seems to be more frequent after bispecific antibodies than CAR T cells due to constant selection pressure with repetitive bispecific antibody application [10–13], and is obviously not the case in our patient albeit not assessed. Molecular analyses to determine reasons for non-response is highly important. However, the clinical reality is that this cannot be elucidated in all patients for all treatment lines yet, but possibly will be in the future, if molecular and genetic testing are reimbursed and performed as standard of care tests to guide MM treatment.

This report suggests that the superiority of cilta-cel over ide-cel extends to patients who received both medications [14]. It shall therefore protect further MM patients from being withheld a possibly effective although still very costly treatment. Our case also suggests quality-of-life improvement in this heavily pretreated patient since no further MM therapy has been required after cilta-cel.

## Data Availability

No datasets were generated or analysed during the current study.
